# Neural Representations of Death in the Cortical Midline Structures Promote Temporal Discounting

**DOI:** 10.1093/texcom/tgab013

**Published:** 2021-02-22

**Authors:** Kuniaki Yanagisawa, Emiko S Kashima, Yayoi Shigemune, Ryusuke Nakai, Nobuhito Abe

**Affiliations:** Department of Psychology, Graduate School of Humanities, Kobe University, Kobe 657-8501 Japan; School of Psychology and Public Health, La Trobe University, Bundoora, Vic 3086, Australia; Research and Development Initiative, Chuo University, Tokyo 192-0393, Japan; Kokoro Research Center, Kyoto University, Kyoto 606-8501, Japan; Kokoro Research Center, Kyoto University, Kyoto 606-8501, Japan

**Keywords:** death-related information, default mode, delay discounting, fMRI, MVPA

## Abstract

Death is an important reminder that our lives are finite. Although some studies have shown that thinking about one’s own death increases temporal discounting (i.e., the devaluing of future rewards), the underlying neural mechanisms are still unknown. In a functional magnetic resonance imaging experiment, we compared the neural and behavioral processes of temporal discounting across four conditions involving distinct types of future thinking (death related, negative, neutral, and positive). Replicating prior research, the behavioral evidence showed that temporal discounting increased when thinking about one’s own future death. Multivoxel pattern analysis showed that death-related future thinking was decoded in default mode regions, including the inferior parietal lobule, precuneus, and medial prefrontal cortex (MPFC). When future thinking was death related (vs. negative), increased temporal discounting was associated with a higher decoding accuracy in the precuneus and MPFC. The present findings suggest that death-related neural representations are distributed across default mode regions, and neural populations in the cortical midline structures play a crucial role in the integration of one's own death into economic decision-making.

## Introduction

While our futures are largely uncertain, there is one guarantee: we will all eventually die. This perception of the finiteness of life could lead individuals not only to the notion that time is limited but also to the realization that the timing of their death is entirely unpredictable. Awareness of the inevitability and unpredictability of death could affect various kinds of psychological processes by increasing concerns about death. For example, behavioral and neuroimaging evidence suggests that thoughts of death modulate reward learning, self-referential processing, and empathy (Li et al. 2015; Chen et al. 2019; Luo et al. 2019).

Thoughts of death can also affect decision-making about the future. One promising way to reveal the association between thinking about death and future-oriented decision-making is to focus on temporal discounting. Temporal discounting is the devaluing of future rewards relative to present rewards. Although there are a variety of factors that contribute to discounting future rewards, the ultimate motive is derived from the fact that a person could die before obtaining his or her future rewards (Story et al. 2015). For example, previous research has found that people are less likely to wait for a future reward after (compared with before) experiencing a disaster (Li et al. 2011) and when they feel their lives are at risk (Chao et al. 2009; Lahav et al. 2011). These studies suggest that death-related information highlights the risk of delaying rewards to the future and thereby produces a higher preference for immediate rewards. Consistent with this perspective, previous studies have found evidence that mortality cues can increase temporal discounting (Griskevicius et al. 2011; Zaleskiewicz et al. 2013).

Can familiar neural mechanisms explain the preferences for immediate rewards after exposure to a reminder of death in the future? A key candidate is the default mode network (DMN; Raichle 2015), comprising regions in the medial temporal lobe, precuneus, medial prefrontal cortex (MPFC), and lateral temporal and parietal regions. The DMN also corresponds to the episodic future thinking network: this brain network functions adaptively to integrate information about relationships and associations from past experiences to construct mental simulations about possible future events (Buckner and Carroll 2007; Schacter et al. 2007, 2017; Spreng et al. 2009). In recent multivariate pattern analyses, neural activity patterns within these regions have been shown to carry information about individual people (Hassabis et al. 2014) and locations (Robin et al. 2018) during the imagining of episodic events. In addition, Satpute and Lindquist (2019) claimed that the DMN plays a constitutive role in creating discrete emotional experiences by drawing on prior experience and knowledge. Thus, given the involvement of the DMN in foresight as well as episodic memory colored with emotion, it seemed plausible that neural activation patterns in the DMN would reflect a person’s simulated, emotional experiences driven by concerns about death.

Among the DMN regions, neural representation in the MPFC is likely to track the probability of acquiring future rewards and thereby to modulate the subjective value of a reward in the present relative to a future context. This hypothesis was inspired by the following two lines of evidence. First, studies have highlighted the key role of the MPFC in subjective valuation processes (Kable and Glimcher 2009; Peters and Buchel 2010; Levy and Glimcher 2012; Bartra et al. 2013). For example, Seaman et al. (2018) examined preferences for effort, probability, and time in monetary decisions across adulthood. Despite preferences for lower physical effort, higher probability, or shorter time delays being uncorrelated, they found overlapping activity associated with subjective valuation in the MPFC. Second, Peters and Buchel (2010) reported that temporal discounting was modulated by imagining one's future activities in detail and that its effect was related to the extent of MPFC activation. These findings are highly consistent with the notion that the MPFC supports the intersection of future thinking and valuation of rewards (Schacter et al. 2017). We therefore predicted that death-related neural representations in the MPFC track a highly reduced probability of reward acquisition at the time of death, which in turn adds weight to the value of rewards in the (immediate) present context.

We tested our predictions by conducting a functional magnetic resonance imaging (fMRI) study involving healthy young adults performing a delay discounting task. In this task, participants were presented with a series of episodic scenarios leading them to imagine their possible future and then were asked to choose between an immediate reward and a delayed reward. Both death-relevant and nondeath-relevant negative situations were included in the scenarios (e.g., Yanagisawa et al. 2016), as well as positive and neutral situations, to separate the effects of death relevance from those of negative situations in assessing temporal discounting. We performed region of interest (ROI)-based multivoxel pattern analysis (MVPA), which allowed us to identify the brain regions that represented death-related information. Compared with conventional univariable analysis, MVPA is more sensitive in detecting fine-grained differences in the spatial patterns of neural activity elicited in different experimental conditions. We examined whether death-relevant information was decoded from the patterns of neural activity in the DMN. In addition, after confirming the effect of thinking about one's own death on increased temporal discounting, we correlated this effect with the decoding accuracy of death-related information in the DMN, especially in the MPFC.

## Material and Methods

### Participants

Thirty healthy, right-handed young adults (16 males and 14 females; age range 20–29 years, *M* = 23.2) with no history of neurological or psychiatric disease participated in this study. The optimal sample size was determined based on a G-Power analysis (Faul et al. 2009) using a power of 0.9, a medium effect size (cf. Benoit et al. 2011; Peters and Buchel 2010) of *f* = 0.25 for repeated-measures analysis of variance (ANOVA) with four measurements and an α level of 0.05; data collection ceased when this number was satisfied. All participants provided written informed consent to participate in this study in accordance with a protocol approved by the ethics committee of Kyoto University.

### Stimuli

All stimuli were presented using Presentation software (Neurobehavioral Systems, USA). The stimuli consisted of 80 short descriptions of possible life episodes, including 20 death-related episodes (e.g., “I was diagnosed with terminal cancer”), 20 negative episodes (e.g., “I was fired by my company”), 20 neutral episodes (e.g., “I submitted my resume”) and 20 positive episodes (e.g., “I met someone I admire”). To validate the stimuli, an independent group of 20 individuals who did not participate in the fMRI study described here rated each episode on an 8-point scale in terms of (1) semantic death relevance (1 = “not related at all” and 8 = “very strongly related”), (2) emotional valence (1 = “extremely positive” and 8 = “extremely negative”), and (3) arousal (1 = “not arousing” and 8 = “very arousing”). The scores for each category were subjected to ANOVA. Significant category effects were found for death relevance, *F*(3,76) = 943.79, *P* < 0.001, η^2^ = 0.97 (Supplementary Fig. 1*A*), emotional valence, *F*(3,76) = 626.56, *P* < 0.001, η^2^ = 0.96 (Supplementary Fig. 1*B*), and arousal, *F*(3,76) = 18.59, *P* < 0.001, η^2^ = 0.42 (Supplementary Fig. 1*C*). Bonferroni-adjusted *P*-values and Tukey-adjusted confidence intervals the for mean difference (MD) were used to evaluate the statistical significance of the post hoc comparisons, which confirmed that the death relevance was greater for the death-related episodes (*M* = 7.29) than for the negative episodes (*M* = 2.62), MD = 4.67 (95% CI, 4.33–5.02), *t*(76) = 35.83, *P* < 0.001, neutral episodes (*M* = 1.44), MD = 5.86 (95% CI, 5.52–6.20), *t*(76) = 44.91, *P* < 0.001, and positive episodes (*M* = 1.23), MD = 6.07 (95% CI, 5.72–6.41), *t*(76) = 46.51, *P* < 0.001. Furthermore, the death relevance was greater for the negative episodes than for the neutral episodes, MD = 1.19 (95% CI, 0.84–1.53), *t*(76) = 9.09, *P* < 0.001, and the positive episodes, MD = 1.39 (95% CI, 1.05–1.74), *t*(76) = 10.68, *P* < 0.001. The emotional valence for the death-related episodes (*M* = 7.09) and negative episodes (*M* = 6.87), MD = 0.22 (95% CI, −0.14 to 0.58), *t*(76) = 1.63, *P* = 0.644, was comparable but greater (i.e., more negative) than for the neutral episodes (*M* = 4.40), MD = 2.70 (95% CI, 2.34–3.06), *t*(76) = 19.76, *P* < 0.001, and MD = 2.48 (95% CI, 2.12–2.83), *t*(76) = 18.13, *P* < 0.001, respectively, and the positive episodes (*M* = 1.95), MD = 5.14 (95% CI, 4.78–5.50), *t*(76) = 37.66, *P* < 0.001, and MD = 4.92 (95% CI, 4.56–5.28), *t*(76) = 36.04, *P* < 0.001, respectively. The arousal levels for the death-related episodes (*M* = 5.83), negative episodes (*M* = 5.73) and positive episodes (*M* = 5.82) were similar, *P*s = 1.00, but greater than for the neutral episodes (*M* = 4.21), MD = 1.62 (95% CI, 0.94–2.30), *t*(76) = 6.23, *P* < 0.001, MD = 1.52 (95% CI, 0.84–2.20), *t*(76) = 5.85, *P* < 0.001, and MD = 1.61 (95% CI, 0.93–2.29), *t*(76) = 6.19, *P* < 0.001, respectively.

### Delay Discounting Task

**Figure 1:**
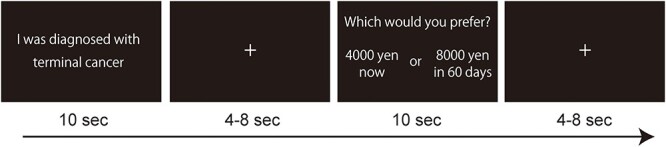
Delay discounting task used in the fMRI experiment. Each trial began with the presentation of a scenario for 10 s; then, the immediate reward (held constant at 4000 yen) and delayed reward (e.g., 8000 yen in 60 days) options were presented for 10 s. The participants indicated their preference for either the immediate or the delayed reward.

During the fMRI scans, the participants completed a delay discounting task (Fig. 1) that was modified from Benoit et al. (2011). First, the participants were told that this study examined the effects of imagining future events on monetary decisions. They were instructed to project themselves into each scenario with as much detail as possible to mentally experience the situation. The scenarios were presented one by one in random order. Each trial began with the presentation of a scenario for 10 s, and then, the immediate reward (held constant at 4000 yen) and delayed reward (e.g., 8000 yen in 60 days) options were presented for 10 s. The participants indicated their preference for the immediate or delayed reward. The amount of the delayed reward varied among five values (4500, 5500, 6500, 8000, and 10 000 yen), and the length of delay varied among four values (30, 60, 180, and 360 days). These parameters were selected based on Benoit et al. (2011). The complete crossing of the reward and delay conditions yielded a total of 20 trials, which were presented in random order. The session was divided into four functional runs, each including 5 trials each of the death-related, negative, neutral, and positive scenarios. The intervals between the scenarios and reward options, during which a cross-shaped fixation was continuously presented, ranged from 4 to 8 s to maximize the efficiency of the event-related design (Dale 1999).

After the scanning session, the participants completed the same delay discounting task as used in the fMRI scan (without imaging future events) to measure the baseline level of temporal discounting. Following this task, the participants were presented with the same scenarios again on the computer screen and asked to rate the vividness of the imagined future events on a scale from 1 (not vivid or clear at all) to 8 (extremely vivid or clear).

### Data Acquisition

Whole-brain imaging was performed using a 3.0-Tesla Magnetom Verio MRI scanner (Siemens, Erlangen, Germany). A T2^*^-weighted echo planar imaging (EPI) sequence sensitive to blood oxygenation level-dependent (BOLD) contrast was used for functional imaging. The following parameters were used: repetition time (TR) = 2500 ms, echo time (TE) = 30 ms, flip angle = 90°, acquisition matrix = 64 × 64, field of view = 224 mm, in-plane resolution = 3.5 × 3.5 mm, number of axial slices = 39, and slice thickness = 3.5 mm. The acquisition sequence was tilted by 30° with respect to the anterior commissure–posterior commissure (AC–PC) line to recover the magnetic susceptibility-induced signal losses caused by the sinus cavities (Deichmann et al. 2003). High-resolution (spatial resolution 1 × 1 × 1 mm) structural images were acquired using a T1-weighted, magnetization-prepared, rapid-acquisition gradient echo (MP-RAGE) pulse sequence. Firm padding was placed around the head of each participant to restrict head motion. The visual stimuli were projected onto a screen and viewed through a mirror that was attached to a standard head coil. The participants’ responses were collected using an MRI-compatible response box. The first four scans in each run were discarded to allow for T1 equilibration effects.

### Data Processing

Data preprocessing and statistical analysis were performed using Statistical Parametric Mapping (SPM)-12 software (Wellcome Department of Imaging Neuroscience, London, UK). All functional images for each participant were corrected for the slice acquisition time. The resulting images were then realigned to correct for small movements that occurred between scans. This process generated an aligned set of images and a mean image for each participant. Each participant’s T1-weighted structural MRI was coregistered to the mean of the realigned EPI images and segmented to isolate the gray matter, which was normalized to the gray matter in a template image based on the Montreal Neurological Institute (MNI) reference brain. Using the parameters of this normalization process, the EPI images were also normalized to the MNI template (resampled voxel size: 2 × 2 × 2 mm).

### Multivariate Classification Analyses

The multivariate classification analyses were performed with the CoSMoMVPA toolbox (Oosterhof et al. 2016) implemented in MATLAB. These analyses were performed using support vector machine (SVM) classifiers as implemented in LIBSVM (Chang and Lin 2011). For the MVPA, we estimated a general linear model (GLM) based on the unsmoothed data to preserve the maximal amount of spatial information. Each experimental condition interval (i.e., onset with the presentation of the scenario, which persisted for 10 s) was modeled using a canonical hemodynamic response function. This GLM included all single-trial regressors (i.e., a total of 80 β images; 20 images [5 death-related trials, 5 negative trials, 5 neutral trials, and 5 positive trials] × 4 runs). Motion parameters (6 regressors for each run) estimated in the realignment procedure were also included in the GLM to regress out potential motion-induced signal fluctuations. A high-pass filter with a frequency of 1/128 Hz was used to remove low-frequency noise, and a first-order autoregressive (AR[1]) model was employed to correct for temporal autocorrelations. The estimated β images from the GLM were used for SVM classification.

We performed ROI-based MVPA to examine the spatial pattern of activity across voxels within the brain regions involved in episodic future thinking. The brain regions that underlie episodic future thinking were defined using a meta-analysis map (association test) of voxels associated with “default mode” from the NeuroSynth online database (http://neurosynth.org; Yarkoni et al. 2011; Supplementary Fig. 2). This ROI (a total of 10 715 voxels) comprises brain regions that have been preferentially implicated in neuroimaging studies that addressed the neural bases of the DMN and includes areas involved in episodic future thinking. From this mask, we then isolated clusters of individual brain regions that included more than 100 voxels and conducted the same ROI-based MVPA. The clusters included the MPFC (2416 voxels), the precuneus (3517 voxels), the bilateral angular gyri (L, 1067 voxels; R, 907 voxels), the bilateral parahippocampal gyri (L, 154 voxels; R, 112 voxels), the bilateral middle temporal gyri (L, 570 voxels; R, 419 voxels), the bilateral superior frontal gyri (L, 279 voxels; R, 583 voxels), and the cerebellum (130 voxels).

A linear SVM classifier as implemented in LIBSVM (Chang and Lin 2011) was trained using the β maps of three runs, and classification was performed for the β map of the remaining run to evaluate the performance of the classifier. This leave-one-run-out cross-validation procedure was repeated for all combinations of runs. Using a linear discriminant analysis (LDA) classifier implemented in the CoSMoMVPA toolbox (Oosterhof et al. 2016), we also replicated the present findings (see Supplementary Information for details and results: Supplementary Tables 3 and 4). The decoding accuracy was computed for each individual in each ROI. For each classification analysis, we used the Bayesian Wilcoxon signed-rank test to determine whether the classification performance was above the chance level. We calculated the Bayes factor (BF), which is the likelihood ratio of the null and alternative hypotheses (e.g., classification performance > 0), using JASP software (JASP Team 2020). We asserted that a BF_10_ less than 0.1 implied strong evidence for the null hypothesis, a BF_10_ between 0.1 and 0.33 provided moderate evidence for the null hypothesis, a BF_10_ between 0.33 and 3 suggested only weak or inconclusive evidence for the hypotheses, a BF_10_ between 3 and 10 denoted moderate evidence for the alternative hypothesis, a BF_10_ between 10 and 30 implied strong evidence, a BF_10_ between 30 and 100 implied very strong evidence and a BF_10_ greater than 100 suggested extreme evidence for the alternative hypotheses (Lee and Wagenmakers 2014).

In addition to the BF, we also performed random permutation tests in each ROI at the single-subject level and then combined the results at the group level with a bootstrap method (Stelzer et al. 2013). For each participant, we trained and tested the classifier repeatedly on data in which the condition labels had been randomly permuted. This process was repeated 100 times, resulting in 100 accuracy values for each participant. From each participant’s permutation accuracy values, one value was randomly chosen and averaged across all of the participants. This process was repeated 10 000 times to generate a distribution of the expected group accuracy under the null hypothesis. The position of the observed group accuracy in this null distribution was used to determine a *P*-value. The *P-*values were Bonferroni corrected for multiple comparisons, adjusting them to the number of ROIs (11) tested.

We tested whether the classifier accuracy in any of the DMN ROIs correlated with either each participant’s (1) vividness of the imagined future death-related events or (2) the effect of thinking about one’s own death on the reward index. For this purpose, we calculated the Spearman correlation coefficients (one-tailed). *P-*values were Bonferroni corrected for multiple comparisons, adjusting them to the number of ROIs (3) tested. The results were visualized in R version 3.5.1 (R Core Team 2019) with RStudio (RStudio Team 2015) using the “ggpubr” (Kassambara 2019) and “ggplot2” (Wickham 2016) packages.

To provide complementary information to the ROI-based MVPA, we subsequently performed a searchlight MVPA (Kriegeskorte et al. 2006) with a radius of 4 voxels. Decoding accuracies from each searchlight were assigned to the central voxel. To identify voxels where the decoding accuracy was greater than chance, we performed a random permutation test (Stelzer et al. 2013), as implemented in CoSMoMVPA (Oosterhof et al. 2016), similar to the procedure we used for the ROI-based analysis. For searchlight MVPA, the observed and null accuracy maps were entered into CoSMoMVPA’s Monte Carlo cluster statistics function, which returned a statistical map corrected for multiple comparisons using threshold-free cluster enhancement (TFCE; Smith and Nichols 2009), yielding a group-level *z*-score map of the classifier results. For visualization purposes, we projected group maps on a segmented and inflated the MNI-aligned brain (Colin Holmes’ 27-scan average brain image, as implemented in NeuroElf, v 1.1) in BrainVoyager (version 21.0, BrainInnovation). In addition, to confirm the brain regions in which the decoding accuracy (death related and negative) was significantly associated with the reward index, multiple regression analysis was performed using the Statistical nonParametric Mapping (SnPM) toolbox (http://nisox.org/Software/SnPM13/) with 5000 permutations. The statistical threshold was set at *P* < 0.05 corrected for multiple comparisons at the cluster level over the search volume (familywise error) with a height (cluster-forming) threshold of *P* < 0.001.

## Results

### Future Thinking About One’s Own Death Facilitates Temporal Discounting

Following Palombo et al. (2015, 2016), the “reward index” was analyzed as a measure of temporal discounting. The reward index reflects the acceptance of delayed rewards or the extent to which an accumulated reward exceeds the amount that would be obtained by always choosing the immediate reward. It is calculated as the difference between the actual accumulated reward and the minimum accumulated reward possible divided by the difference between the maximum accumulated reward possible and the minimum accumulated reward possible. Trials without a response were omitted from the calculation of this index. Therefore, the value of the reward index ranged from 0 to 1.0 as follows: a reward index of 0 indicated the consistent selection of the smaller, immediate reward, while the consistent selection of the larger, future reward resulted in a reward index of 1.0.

**Figure 2:**
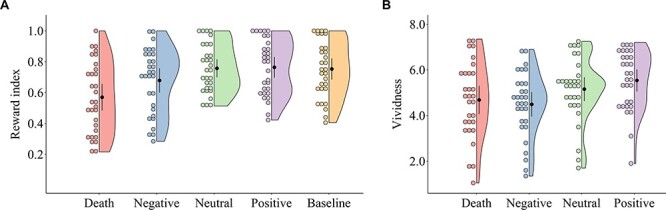
Half-violin plots of the values of (*A*) the reward index and (*B*) the vividness in each condition. The distributions of the reward index and vividness are represented by the outer shape. The black circles represent the mean values; the whiskers represent 95% confidence intervals; and the colored circles represent individual data points. The values of the reward index ranged from 0 (the consistent selection of the smaller, immediate reward) to 1.0 (the consistent selection of the larger, delayed reward).

Repeated-measures ANOVA of the reward index revealed a significant main effect of condition, *F*(4,116) = 11.19, *P* < 0.001, η^2^ = 0.28 (Fig. 2*A*). Bonferroni-adjusted *P*-values and confidence intervals for MD were used to evaluate the statistical significance of the post hoc comparisons. As expected, the reward index was lower for death-related episodes (*M* = 0.57) than for negative episodes (*M* = 0.68), MD = −0.11 (95% CI, −0.21 to −0.01), *t*(29) = −3.11, *P* = 0.024, neutral episodes (*M* = 0.76), MD = −0.19 (95% CI, −0.29 to −0.09), *t*(29) = −5.35, *P* < 0.001, positive episodes (*M* = 0.76), MD = −0.19 (95% CI, −0.29 to −0.10), *t*(29) = −5.54, *P* < 0.001, and baseline (*M* = 0.75), MD = −0.18 (95% CI, −0.28 to −0.08), *t*(29) = −5.23, *P* < 0.001. Therefore, the participants exhibited greater temporal discounting when thinking about death than when thinking about nondeath-related future episodes.

### Vividness of Imagined Future Events

The vividness scores were assessed with repeated-measures ANOVA. This analysis revealed a significant main effect of condition, *F*(3,87) = 7.89, *P* < 0.001, η^2^ = 0.21 (Fig. 2*B*). The vividness of the death-related episodes (*M* = 4.69) and vividness of the negative episodes (*M* = 4.49), MD = 0.19 (95% CI, −0.45 to 0.84), *t*(29) = 0.81, *P* = 1.00, were comparable but lower than those of the positive episodes (*M* = 5.54), MD = −0.85 (95% CI, −1.50 to −0.21), *t*(29) = −3.59, *P* = 0.003, and MD = −1.05 (95% CI, −1.69 to −0.41), *t*(29) = −4.40, *P* < 0.001, respectively. There was no significant difference in vividness between the death-related episodes and the neutral episodes (*M* = 5.16), MD = −0.47 (95% CI, −1.12 to 0.17), *t*(29) = −1.99, *P* = 0.298.

We subsequently examined whether the perceived vividness of the imagined future death-related events was related to the effect of thinking about death on the reward index (i.e., the reward index for the negative episodes minus that for the death-related episodes, where positive values meant greater discounting on the death-related episodes condition than on the negative episodes condition). To adjust for the confounding effects of the vividness of imagined future negative events, we performed a partial correlation analysis. We observed a significant positive correlation, Spearman’s ρ = 0.39, *P* = 0.017 (one-tailed). This result suggested that the participants reporting more vivid imagery for future death-related events exhibited a greater effect of death thoughts on the reward index.

### DMN Regions Represent Death-related Information

**Figure 3:**
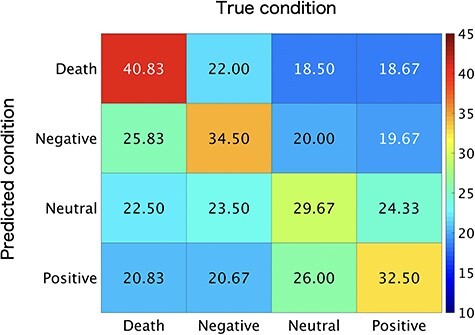
Confusion matrix for the four experimental conditions in the ROI associated with “default mode” from the NeuroSynth online database.

**Figure 4:**
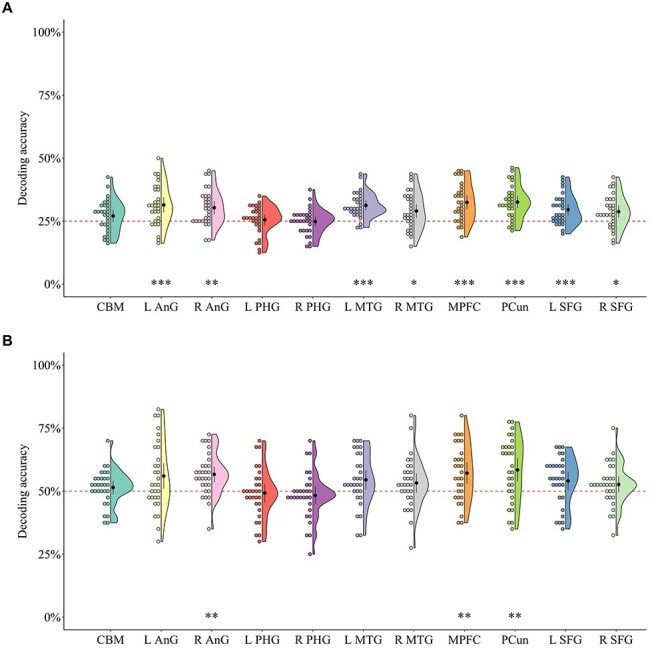
Half-violin plots of the classification performance for (*A*) multiple classes (death related, negative, neutral, and positive) and (*B*) two classes (death related and negative) in each ROI. The distribution of the classification performance is represented by the outer shape. The black circles represent the mean values; the whiskers represent 95% confidence intervals; the colored circles represent individual data points; and the red dashed line represents chance level. CBM, cerebellum; L AnG, left angular gyrus; R AnG, right angular gyrus; L PHG, left parahippocampal gyrus; R PHG, right parahippocampal gyrus; L MTG, left middle temporal gyrus; R MTG, right middle temporal gyrus; MPFC, medial prefrontal cortex; PCun, precuneus; L SFG, left superior frontal gyrus; R SFG, right superior frontal gyrus. ^*^P < 0.05, ^*^^*^P < 0.01, ^*^^*^^*^P < 0.001.

We first performed a multiclass (i.e., death-related, negative, neutral, and positive) MVPA by extracting multivoxel activity patterns in each ROI. We confirmed that the entire DMN ROI based on NeuroSynth showed an above chance-level (i.e., 25%) classification performance (*M* = 34.38, BF_10_ > 100, *P* < 0.001). To better scrutinize the classification performance, we examined the relationship between the predicted and true condition categories. Examination of the confusion matrix suggested that all four types of conditions were successfully classified (Fig. 3). In addition, we found that 8 (of 11) ROIs, including the bilateral angular gyri, the bilateral middle temporal gyri, the MPFC, the precuneus, and the bilateral superior frontal gyri ROIs, showed above chance-level classification performance (Supplementary Table 1; Fig. 4*A*). Bayesian analyses showed strong-to-extreme evidence in favor of the alternative hypothesis in the bilateral angular gyri, the left middle temporal gyrus, the MPFC, the precuneus, and the left superior frontal gyrus.

To further identify the brain regions that specifically represented death-related information, we performed two-class (i.e., death-related and negative) MVPA. Consistent with our hypothesis, the entire DMN ROI based on NeuroSynth showed above chance-level (i.e., 50%) classification performance (*M* = 60.33, BF_10_ > 100, *P* < 0.001). In addition, we found that three (of 11) ROIs, including the right angular gyrus, the MPFC, and the precuneus ROIs, showed above chance-level classification performance (Supplementary Table 2; Fig. 4*B*). Bayesian analyses showed very strong-to-extreme evidence in favor of the alternative hypothesis in these regions.

 We subsequently examined whether individual differences in classification performance in the above three ROIs (right angular gyrus, MPFC, precuneus) were related to the vividness of the imagined future death-related events. To adjust for the confounding effects of the vividness of imagined future negative events, we performed a partial correlation analysis. Positive significant correlations were found in the right angular gyrus and the precuneus (right angular gyrus: ρ = 0.46, *P* = 0.018; MPFC: ρ = 0.30, *P* = 0.168; precuneus: ρ = 0.53, *P* = 0.006). These results suggested that participants who showed more vivid imagery for future events exhibited different neural activity patterns of death-related and negative events across default mode regions.

**Figure 5:**
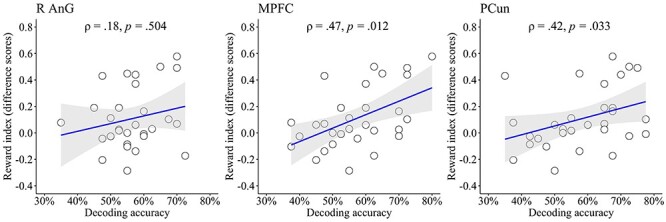
Correlations between the individual differences in the effect of thinking about one’s own death on the reward index and the decoding accuracies. The shaded areas reflect the 95% confidence intervals. The *P*-value displayed is Bonferroni corrected for multiple comparisons. R AnG, right angular gyrus; MPFC, medial prefrontal cortex; PCun, precuneus.

Furthermore, we also examined whether individual differences in classification performance in the above three ROIs predicted the effect of thinking about one’s own death on the reward index (i.e., the reward index for the negative episodes minus the equivalent for the death episodes). Positive significant correlations were found in the MPFC and the precuneus (right angular gyrus: ρ = 0.18, *P* = 0.504; MPFC: ρ = 0.47, *P* = 0.012; precuneus: ρ = 0.42, *P* = 0.033, Fig. 5). These results suggested that death-related neural representations were distributed across the default mode regions and that the neural populations in the MPFC and the precuneus induced a shift towards more present-oriented choices.

### Searchlight MVPA

**Figure 6:**
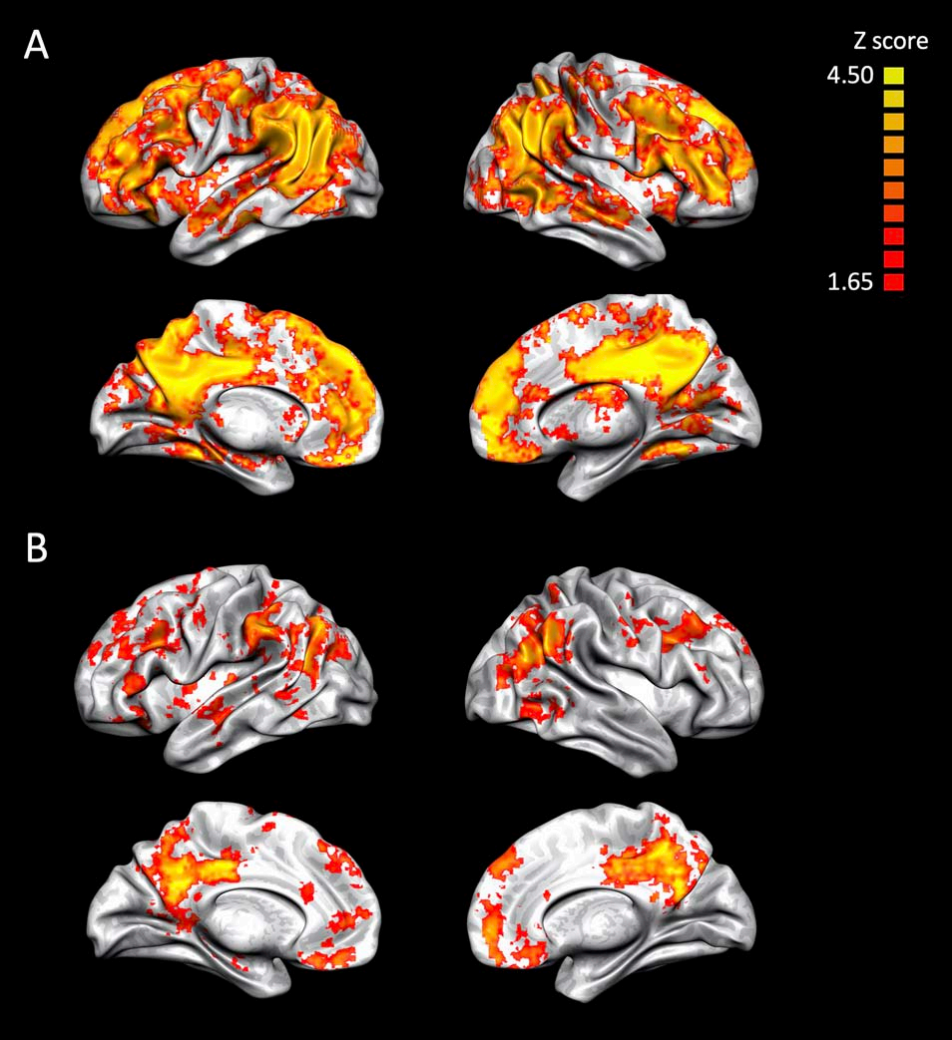
Results of the searchlight analyses at the group level. Brain regions that showed above chance-level classification performances in the (*A*) multiclass (death related, negative, neutral, and positive) and (*B*) two-class (death related and negative) analyses. The threshold was set at a TFCE-corrected *P* < 0.05 (one-tailed, *z* = 1.65).

To complement the results of the ROI-based MVPA, we performed a whole-brain searchlight MVPA to identify brain areas that locally represented death-related information. We first performed a multiclass (i.e., death related, negative, neutral, and positive) searchlight MVPA. This analysis replicated the results of the ROI-based MVPA: local activity patterns in the DMN regions showed above chance-level classification performance (Fig. 6*A*). In addition to these areas, the searchlight MVPA identified clusters in the lateral prefrontal cortex. To further identify the brain regions that specifically represented death-related information, we performed a two-class (i.e., death and negative) searchlight MVPA. This analysis also replicated the results of the ROI-based MVPA: local activity patterns in the DMN regions carried death-related information (Fig. 6*B*).

Finally, to confirm the brain regions in which the decoding accuracy (death related and negative) was significantly associated with the reward index, multiple regression analysis was performed. This analysis replicated the results of the ROI-based analysis: a positive significant correlation was found in the MPFC (peak MNI coordinates *x* = 12, *y* = 52, *z* = 34) and the precuneus (peak MNI coordinates *x* = 14, *y* = −36, *z* = 38) (see Supplementary Fig. 3).

## Discussion

In the present study, fMRI was utilized to investigate the interactions between thinking about one’s own future death and intertemporal decision-making. Consistent with previous findings, the behavioral data showed that temporal discounting increased when thinking about one’s own future death. The neuroimaging data demonstrated that the DMN regions, including the angular gyrus, the precuneus and the MPFC, represented death-related information. In addition, individual differences in classification performance in the precuneus and the MPFC were correlated with the effect of thinking about one’s own death on temporal discounting. To the best of our knowledge, the present results are the first to demonstrate that death-related information is represented across DMN regions and that cortical midline structures play a crucial role in promoting present-oriented decisions.

The primary finding of the present study was that the DMN regions, especially the angular gyrus, the precuneus and the MPFC, represent not only emotional information but also death-related information. Previous studies have shown that these neural regions are critical in creating multiple emotional experiences by drawing on prior experience and knowledge (Satpute and Lindquist 2019). Expanding on these previous studies, the present study shed further light on the neural architectures responsible for imagining future death in the DMN regions. Given that death-related events are reminders of notions such as inevitability and unpredictability, the higher classification performance in these regions was likely due to the existential fear of death aroused in the participants. This is consistent with our findings showing that individual differences in classification performance in the above regions, especially the precuneus, a region implicated in mental imagery (Buckner and Carroll 2007; Schacter et al. 2007, 2017; Spreng et al. 2009), correlated with the vividness of the imagined future death-related events. In addition, the present findings provide new insights into the diversity observed in previous neuroimaging studies of death-related thoughts. Past fMRI studies using standard univariate fMRI analysis have shown that the regions recruited by death-related thoughts are not consistent across studies (e.g., Han et al. 2010; Quirin et al. 2012; Shi and Han 2013). The MVPA approach used in the present study directly captures fine-grained spatial patterns that can discriminate between experimental conditions (Haynes 2015; Norman et al. 2006) and is thus particularly suitable for the identification of brain regions that represent complex psychological processes, including death-related thoughts.

Importantly, we demonstrated that the neural representation in the cortical midline structures may play a direct role in an episodic future thought impacting temporal discounting by modulating the relative weights given to the reward values in the present and future contexts. More specifically, it appears that the MPFC tracks a highly reduced probability of reward acquisition at the time of death and adds weight to the immediate reward (i.e., increased temporal discounting). A previous fMRI study reported that temporal discounting was reduced by imagining one's future activities in detail and that the extent of the effect was associated with MPFC activation (Peters and Buchel 2010). Initially, their behavioral finding seems to contradict our results, but the nature of the future events imagined in the task is different between the two studies. Peters and Buchel (2010) used neutral or positive events for participants’ future plans that are conceivable and desirable, whereas we used death-related events that elicit existential fear. Our interpretation here, therefore, reconciles these two lines of evidence: the MPFC “flexibly” modulates the relative weights of the reward values in present and future contexts in a manner critically dependent on the probability of reward acquisition at the time of the imagined events.

We speculate that the increased temporal discounting is an adaptive consequence of imagining future death rather than a negative consequence or unwanted side effect. Typically, temporal discounting has been regarded as a reliable marker for impulsivity (Bickel et al. 2014). For example, drug-dependent individuals discount delayed reinforcers more rapidly than individuals who are not drug dependent (Bickel et al. 2012). Temporal discounting is also known to strengthen the decision to engage in maladaptive health and financial behaviors (Snider et al. 2019). However, we emphasize that the participants’ preferences for immediate rewards, temporarily enhanced by imagining future death, were likely based on calculated, deliberative processes rather than impulsive, spontaneous processes. As argued by Story et al. (2015), death creates a fundamental motive not to defer rewards for too long, and in computational terms, death can be considered an absorbing state from which no future reward can be harvested. Thus, when confronted with opportunities to imagine future death, the preferential shift to immediate rewards, rather than future rewards, is likely to be a highly adaptive and reasonable response.

An alternative explanation of the present findings is that self-regulation can be temporarily impaired by situational factors, such as mortality salience. Previous studies have supported the view that a preoccupation with thoughts of death depletes self-regulatory resources (Gailliot et al. 2006), allowing impulsive processes to have an increased impact on behavior (Friese and Hofmann 2008). In particular, temporal discounting has been considered pivotal in determining the level of impulsivity in intertemporal choice scenarios (Green and Myerson 2004). Therefore, our results could be interpreted as the depletion of self-control due to an aggressive suppression of thoughts about death. However, this explanation is unlikely because no significant correlation was found between the reward index and the decoding accuracy (death related and negative) in brain regions associated with the regulation and suppression of thoughts, including the dorsolateral prefrontal cortex (Supplementary Fig. 3) (Kikuchi et al. 2010; Benoit and Anderson 2012; Anderson and Hanslmayr 2014; Gagnepain et al. 2014; Kikuchi and Abe 2017), when the participants thought about their own deaths. In addition, the behavioral data showed that those who imagined the death-related events more vividly exhibited a greater effect of thinking about death on temporal discounting. Therefore, we tentatively suggest that our data are not consistent with this alternative idea, although more studies are needed to reach a definitive conclusion.

One limitation of the present study is that we were unable to analyze the neural basis of temporal discounting because the number of trials for the delay discounting task (i.e., 20 trials for each condition) was not sufficient. In particular, the 20 trials would need to be further subdivided into trials with immediate-reward responses and those with delayed-reward responses. Thus, we can use only a small number of trials for each condition to analyze differences in the patterns of brain activity between immediate- and delayed-reward responses. Future studies should address this issue using a design optimized to examine how death-related neural representations modulate the neural basis of temporal discounting.

## Conclusions

The Roman poet Horace once wrote “Carpe diem, quam minimum credula postero,” which can be translated as “seize the day, putting as little trust as possible in the future.” As indicated by Horace, death is a reminder that our lives are finite. It is therefore not surprising that thoughts of death make a person more inclined towards things highly valued for today over the future. The present study provides a neural explanation for this unique, adaptive, and human-specific valuation mechanism: within the DMN, which represents death-related information, the cortical midline structures add weights to reward values in a present context.

## Supplementary Material

Supplementary_tgab013

## Data Availability

The data that support the findings of this study are available on request from the corresponding author, K.Y.

## Notes

This work was conducted using an MRI scanner and related facilities at the Kokoro Research Center of Kyoto University. *Conflict of Interest*: None declared.
