# Supplementary material for: Neural Representations of Death in the Cortical Midline Structures Promote Temporal Discounting
Source: Cereb Cortex Commun. 2021 Feb 22;2(2):tgab013. doi: 10.1093/texcom/tgab013 (PMC8152905; doi:10.1093/texcom/tgab013)
Supplement: Supplementary_tgab013 [file supplementary_tgab013.docx]

Supplementary online materials for “Neural representations of death in the cortical midline structures promote temporal discounting”

Kuniaki Yanagisawa, Emiko S. Kashima, Yayoi Shigemune, Ryusuke Nakai, Nobuhito Abe.

**Stimuli**

**Death-related episodes**

1. I was told by a doctor how long I could live
2. I became unconscious and in critical condition after an accident
3. I was infected with a highly lethal virus
4. I was involved in an earthquake disaster
5. I am an elderly person living alone and suffering from severe pneumonia
6. I accidentally fell from an apartment building
7. I was swallowed by a tsunami
8. I am getting old and my life may end soon
9. I failed to escape from a high-rise building fire
10. Steel bars fell and crushed me at a construction site
11. I collided head-on with an oncoming car while driving
12. I fell into a coma due to illness
13. I received life-prolonging treatment
14. I was taken to the hospital because of heart disease
15. I was diagnosed with terminal cancer
16. I have become too old to sustain my life
17. I was diagnosed with leukemia
18. I have become bedridden because of illness
19. I was caught in an earthquake fire
20. I was swallowed by a pyroclastic flow

**Negative episodes**

1. I was harassed at work
2. I lost an envelope with a large amount of money
3. I owe a large debt
4. My spouse asked me for a divorce
5. I failed an important examination
6. I was ostracized at work
7. I was fired by my company
8. The company I work for went bankrupt
9. I committed a crime
10. My spouse cheated on me
11. I received a summons from court
12. I was betrayed by my subordinate in my office
13. I was late for an important meeting
14. My family relationship has deteriorated
15. My house was burglarized
16. I was falsely accused
17. I was deceived by my friend
18. I was forced to quit my favorite job
19. I took over my family's debt
20. My company has not paid my salary

**Neutral episodes**

1. I gave my business card to someone I do not know
2. I looked after my friend's children
3. I gave otoshidama (New Year's money) to my cousin's children
4. I submitted my resume
5. I prepared the meeting documents for the office
6. I drove my child to school
7. I gave an oseibo (end of the year gift) at the end of the year
8. I sent a New Year's card to my boss
9. I trained my subordinates at the office
10. I helped my child with his homework
11. I looked for a new job
12. I opened a bank account
13. I received a baggage inspection at the airport
14. I jogged early in the morning
15. I requested a mail order catalog
16. I answered the Census
17. I packed things for a move
18. I got a copy of my family register
19. I performed gardening at my home
20. I introduced my acquaintance to my boss

**Positive episodes**

1. I took a long vacation and got absorbed in a hobby
2. I got a big pay raise
3. I was promoted at work
4. I was asked out by the person I like
5. I won the lottery
6. I have a happy marriage life
7. I took an epic round-the-world trip
8. I met someone I admire
9. I married the person I love
10. My long-awaited baby was born
11. I went on a honeymoon
12. I achieved great success in my carrier
13. I got a big win at a horse race
14. I was commended for my work
15. I traveled through the country for my vacation
16. I work for a well-paid company
17. My dream came true
18. I celebrated my child's birthday
19. I received a retirement allowance
20. I celebrated my wedding anniversary with my spouse

**
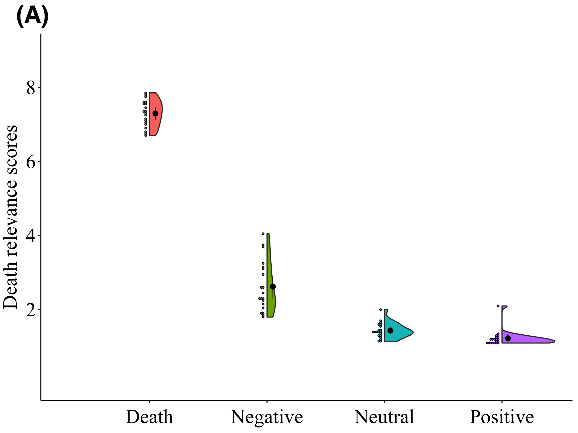
**

**
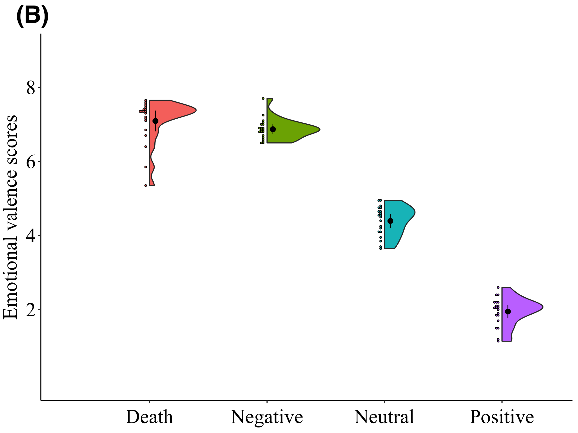
**

**
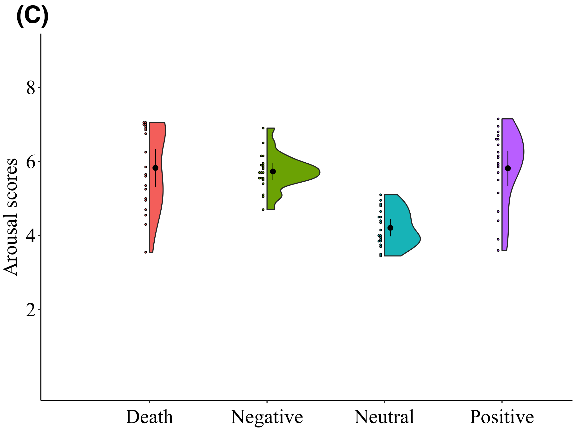
**

**Fig. S1.** Half-violin plots of the scores of (A) death relevance, (B) emotional valence and (C) arousal for the stimuli used in each condition. The distributions of the scores are represented by the outer shape. The black circles represent the mean values; the whiskers represent 95% confidence intervals; and the colored circles represent individual data points.

**
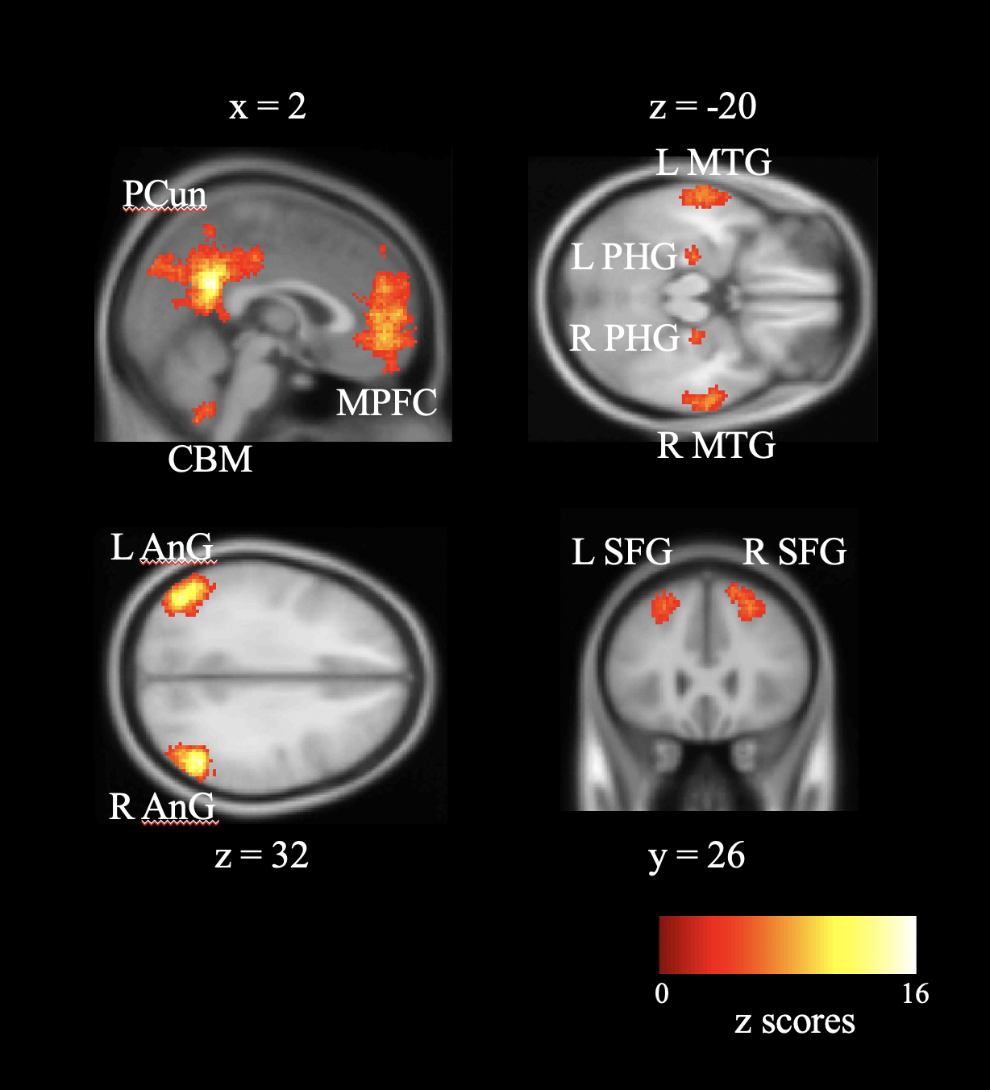
**

**Fig. S2**. Meta-analytic maps show brain regions that are preferentially related to the prespecified search term “default mode”. All regions of interest (ROIs) were visualized using the xjView toolbox (http://www.alivelearn.net/xjview).

CBM, cerebellum; L AnG, left angular gyrus; R AnG, right angular gyrus; L PHG, left parahippocampal gyrus; R PHG, right parahippocampal gyrus; L MTG, left middle temporal gyrus; R MTG, right middle temporal gyrus; MPFC, medial prefrontal cortex; PCun, precuneus; L SFG, left superior frontal gyrus; R SFG, right superior frontal gyrus


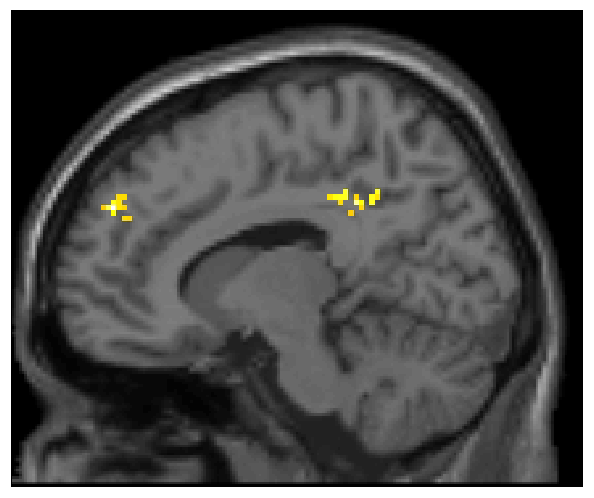


x = 12

**Fig. S3**. Results of the whole-brain group-level correlation analysis. Brain regions in which the decoding accuracy (death-related and negative) was significantly associated with the reward index (i.e., the reward index for the negative episodes minus that for the death episodes) are highlighted.

***Representation of death-related information in DMN regions revealed by a linear discriminant analysis (LDA) classifier***

We performed multiclass (i.e., death-related, negative, neutral, and positive) multivoxel pattern analysis (MVPA) by extracting multivoxel activity patterns in each region of interest (ROI). We determined that seven (of 11) ROIs, including the bilateral angular gyri, the left middle temporal gyri, the MPFC, the precuneus, and the bilateral superior frontal gyri ROIs, showed above chance-level classification performances (Table S3). Bayesian analyses showed extreme evidence in favor of the alternative hypothesis in the bilateral angular gyri, the left middle temporal gyrus, the MPFC, and the precuneus.

To further identify the brain regions that specifically represented death-related information, we performed two-class (i.e., death and negative) MVPA. We determined that five (of 11) ROIs, including the bilateral angular gyrus, the left middle temporal gyrus, the MPFC, and the precuneus ROIs, showed above chance-level classification performance (Table S4). Bayesian analyses showed strong-to-extreme evidence in favor of the alternative hypothesis in these regions.

We examined whether individual differences in classification performance in the above five ROIs were related to the vividness of the imagined future death-related events. To adjust for the confounding effects of the vividness of imagined future negative events, we performed a partial correlation analysis. A positive significant correlation was found in the left angular gyrus and the precuneus (left angular gyrus: ρ = .49, *p* = .015; right angular gyrus: ρ = .33, *p* = .190; left middle temporal gyrus: ρ = .13, *p* = 1.000; MPFC: ρ = .25, *p* = .485; precuneus: ρ = .48, *p* = .020).

We also examined whether individual differences in classification performance in the five ROIs predicted the effect of thinking about one’s own death on the reward index (i.e., the reward index for the negative episodes minus that for the death episodes). A positive significant correlation was found in the MPFC (left angular gyrus: ρ = .36, *p* = .135; right angular gyrus: ρ = .22, *p* = .610; left middle temporal gyrus: ρ = .24, *p* = .500; MPFC: ρ = .49, *p* = .015; precuneus: ρ = .37, *p* = .110). These results largely replicated our main findings using a linear support vector machine (SVM) classifier.

| **Table S1. Results of the multiclass (i.e., death-related, negative, neutral, and positive) ROI-based MVPA.** | | | |
| --- | --- | --- | --- |
| **ROI** | **M** | **BF_10_** | ***p*** |
|  |  |  |  |
| Cerebellum | 0.27 | 1.87 | 0.367 |
| Lt. angular gyrus | 0.32 | 474.07 | 0.001 |
| Rt. angular gyrus | 0.30 | 339.09 | 0.001 |
| Lt. parahippocampal gyrus | 0.26 | 0.34 | 0.500 |
| Rt. parahippocampal gyrus | 0.25 | 0.19 | 0.500 |
| Lt. middle temporal gyrus | 0.31 | 1111.34 | 0.000 |
| Rt. middle temporal gyrus | 0.29 | 20.78 | 0.032 |
| Medial prefrontal cortex | 0.33 | 1307.43 | 0.000 |
| Precuneus | 0.33 | 10691.54 | 0.000 |
| Lt. superior frontal gyrus | 0.30 | 346.51 | 0.001 |
| Rt. superior frontal gyrus | 0.29 | 91.91 | 0.017 |
|  |  |  |  |
| Lt, left; Rt, right |  |  |  |
| *p-*values are corrected for the number of ROIs. | |  |  |

| **Table S2. Results of the two-class (death-related and negative) ROI-based MVPA.** | | | |
| --- | --- | --- | --- |
| **ROI** | **M** | **BF_10_** | ***p*** |
|  |  |  |  |
| Cerebellum | 0.52 | 0.70 | 0.500 |
| Lt. angular gyrus | 0.56 | 4.69 | 0.138 |
| Rt. angular gyrus | 0.57 | 303.76 | 0.001 |
| Lt. parahippocampal gyrus | 0.49 | 0.14 | 0.500 |
| Rt. parahippocampal gyrus | 0.48 | 0.10 | 0.500 |
| Lt. middle temporal gyrus | 0.55 | 9.35 | 0.138 |
| Rt. middle temporal gyrus | 0.53 | 2.52 | 0.456 |
| Medial prefrontal cortex | 0.57 | 66.63 | 0.010 |
| Precuneus | 0.58 | 157.84 | 0.004 |
| Lt. superior frontal gyrus | 0.54 | 5.31 | 0.138 |
| Rt. superior frontal gyrus | 0.53 | 2.33 | 0.456 |
|  |  |  |  |
| Lt, left; Rt, right |  |  |  |
| *p-*values are corrected for the number of ROIs. | |  |  |

| **Table S3. Results of the multiclass (i.e., death-related, negative, neutral, and positive) ROI-based MVPA using a linear discriminant analysis (LDA) classifier.** | | | |
| --- | --- | --- | --- |
| **ROI** | **M** | **BF_10_** | ***p*** |
|  |  |  |  |
| Cerebellum | 0.27 | 0.97 | 0.500 |
| Lt. angular gyrus | 0.30 | 864.93 | 0.000 |
| Rt. angular gyrus | 0.29 | 541.96 | 0.000 |
| Lt. parahippocampal gyrus | 0.26 | 0.37 | 0.500 |
| Rt. parahippocampal gyrus | 0.25 | 0.30 | 0.500 |
| Lt. middle temporal gyrus | 0.30 | 1079.06 | 0.000 |
| Rt. middle temporal gyrus | 0.26 | 0.38 | 0.500 |
| Medial prefrontal cortex | 0.32 | 3589.20 | 0.000 |
| Precuneus | 0.33 | 790.42 | 0.000 |
| Lt. superior frontal gyrus | 0.28 | 47.41 | 0.012 |
| Rt. superior frontal gyrus | 0.29 | 46.56 | 0.012 |
|  |  |  |  |
| Lt, left; Rt, right |  |  |  |
| *p-*values are corrected for the number of ROIs. | |  |  |

| **Table S4. Results of the two-class (death-related and negative) ROI-based MVPA using a linear discriminant analysis (LDA) classifier.** | | | |
| --- | --- | --- | --- |
| **ROI** | **M** | **BF_10_** | ***p*** |
|  |  |  |  |
| Cerebellum | 0.52 | 0.69 | 0.500 |
| Lt. angular gyrus | 0.57 | 20.18 | 0.035 |
| Rt. angular gyrus | 0.57 | 660.55 | 0.004 |
| Lt. parahippocampal gyrus | 0.49 | 0.13 | 0.500 |
| Rt. parahippocampal gyrus | 0.48 | 0.10 | 0.500 |
| Lt. middle temporal gyrus | 0.55 | 21.83 | 0.048 |
| Rt. middle temporal gyrus | 0.54 | 7.94 | 0.137 |
| Medial prefrontal cortex | 0.57 | 56.16 | 0.007 |
| Precuneus | 0.58 | 93.97 | 0.007 |
| Lt. superior frontal gyrus | 0.54 | 3.87 | 0.270 |
| Rt. superior frontal gyrus | 0.53 | 1.50 | 0.500 |
|  |  |  |  |
| Lt, left; Rt, right |  |  |  |
| *p-*values are corrected for the number of ROIs. | |  |  |
